# Precision Medicine in Targeted Therapies for Severe Asthma: Is There Any Place for “Omics” Technology?

**DOI:** 10.1155/2018/4617565

**Published:** 2018-06-11

**Authors:** Carla Galeone, Chiara Scelfo, Francesca Bertolini, Marco Caminati, Patrizia Ruggiero, Nicola Facciolongo, Francesco Menzella

**Affiliations:** ^1^Department of Medical Specialties, Pneumology Unit, Arcispedale Santa Maria Nuova, Azienda USL di Reggio Emilia-IRCCS, Viale Amendola 2, 42122 Reggio Emilia, Italy; ^2^Department of Bio and Health Informatics, Technical University of Denmark, 2800 Kgs. Lyngby, Denmark; ^3^Asthma Center and Allergy Unit, Verona University Hospital, Piazzale L.A. Scuro, 37134 Verona, Italy

## Abstract

According to the current guidelines, severe asthma still represents a controversial topic in terms of definition and management. The introduction of novel biological therapies as a treatment option for severe asthmatic patients paved the way to a personalized approach, which aims at matching the appropriate therapy with the different asthma phenotypes. Traditional asthma phenotypes have been decomposing by an increasing number of asthma subclasses based on functional and physiopathological mechanisms. This is possible thanks to the development and application of different omics technologies. The new asthma classification patterns, particularly concerning severe asthma, include an increasing number of endotypes that have been identified using new omics technologies. The identification of endotypes provides new opportunities for the management of asthma symptoms, but this implies that biological therapies which target inflammatory mediators in the frame of specific patterns of inflammation should be developed. However, the pathway leading to a precision approach in asthma treatment is still at its beginning. The aim of this review is providing a synthetic overview of the current asthma management, with a particular focus on severe asthma, in the light of phenotype and endotype approach, and summarizing the current knowledge about “omics” science and their therapeutic relevance in the field of bronchial asthma.

## 1. Introduction

Severe asthma management still represents a matter of debate, due to asthma heterogeneity and complexity. Today, asthma is classified and assessed according to both phenotypes and endotypes approaches. The last reflects the complex interaction of inflammatory molecules and multiple pathways and systems that are involved in the pathogenesis of asthma. The use of omics technologies represents an effective way for better exploring and defining asthma endotypes. More in general, the omics approach and the application of systems biology methods provide unbiased tools allowing for better understanding of asthma pathophysiology and for developing “precision medicine” approaches. In contrast with the more general but still used “one size fits all” approach, precision medicine consider a specifically targeted therapy that includes specific biological profiles together with patient's exposure and lifestyle. The omics technologies are contributing to the identification of new biomarkers that compose these biological profiles and consequently to the development of targeted biological therapies. For a decade, omalizumab has been the only available therapy for severe allergic asthma. Recently, new promising drugs such as mepolizumab and reslizumab have been introduced as a targeted treatment option for Eosinophilic asthma.

The aim of this review is to offer an overview regarding the management of asthma, from phenotype to inflammatory endotypes. We will focus on pathophysiology mechanisms of severe asthma and on new treatment options based on different endotypes. We will finally discuss the current knowledge about “omics” science and its relevance in exploring new biological endotypes, which will represent the basis for the development of new promising asthma therapies. The transition from new biomarkers discovery and understanding and the development of new successful therapies is still very difficult. Our review will help the clinician to understand how it will be possible to improve the management of severe asthma thanks to the most advanced research tools and what to do to optimize what is already available.

### 1.1. Data Collection Strategy

For this review, a highly sensitive search strategy has been developed, and validated keywords filters have been applied to retrieve articles pertaining to severe asthma definition and management.

In particular a selective search on PubMed and Medline was carried out, and research papers, international guidelines, recommendations, position papers, systematic reviews, and Cochrane meta-analyses relevant to the topic have been included in the review.

We applied a search strategy for identifying the following keywords.

Keywords for part 1 are as follows: asthma phenotypes, asthma endotypes, T2-low and T2-high subtypes, targeted therapies and bronchial thermoplasty coupled with severe asthma.

Keywords for part 2 are as follows: genomics, pharmacogenomics, transcriptomics, epigenomics, proteomics and metabolomics coupled with severe asthma

To retrieve international and European large-scale projects hand searches were performed of the reference lists of all pertinent reviews and studies examined. Abstracts from relevant conferences were searched.

## 2. External Phenotypes and Endotypes

A phenotype is defined as the set of an organism's observable characteristics or traits, such as its morphology and development. As a basic definition, the phenotype is mainly influenced by the interactions between genomic asset and the influence of several environmental factors. At a molecular level, the phenotype is the outcome of the expression and interaction of different endotypes, which are defined by a distinct functional or pathophysiological mechanism [1].

*External Phenotypes of Asthma. *Asthma symptoms are traditionally defined by shortness of breath, wheeze, chest tightness, and cough. However, it is well known that there are different asthma phenotypes. Historically, bronchial asthma was classified as allergic (extrinsic) or nonallergic (intrinsic). Extrinsic atopic asthma generally develops under the age of 40, and it is triggered by inhaled allergens and is usually associated with other allergic diseases, such as rhinitis and dermatitis [2]. On the other hand, intrinsic asthma typically develops later in life (>40 years old) and is usually less recognizable. By definition, intrinsic asthma is not associated with allergic sensitization, but aspirin-intolerance often triggers disease exacerbations. Nowadays, asthma phenotyping also includes several clinical information such as age, concomitant comorbidities (obesity, allergic rhinitis, and sinusitis), exacerbations factors (exercise, allergens, and infections), and response to the treatment.

*Endotypes. *An endotype is specifically defined by the pathophysiologic mechanisms underlying the phenotype(s). The management of severe asthma is benefitting from the characterization of an increasing number of different endotypes, which represent the targets of specific therapies [3].

## 3. T2 Subtypes

Asthma phenotyping based on inflammatory cell count (Eosinophilic, Neutrophilic, and Paucigranulocytic) in tissue and blood is gaining an increasing interest. Nowadays, two main subtypes of type 2 inflammation have been defined: T2-high (T helper type 2 cell high) and T2-low (T helper type 2 cell low) [4, 5].

The T2-high subtype is characterized by the presence of high eosinophil level in airways and includes the following: 1. early onset, allergic sensitization, responsiveness to inhaled corticosteroids (ICS); 2. late-onset, absence of allergic sensitizations, sinusitis, and lack of ICS responsiveness; 3. exercised-induced asthma.

**Figure 1:**
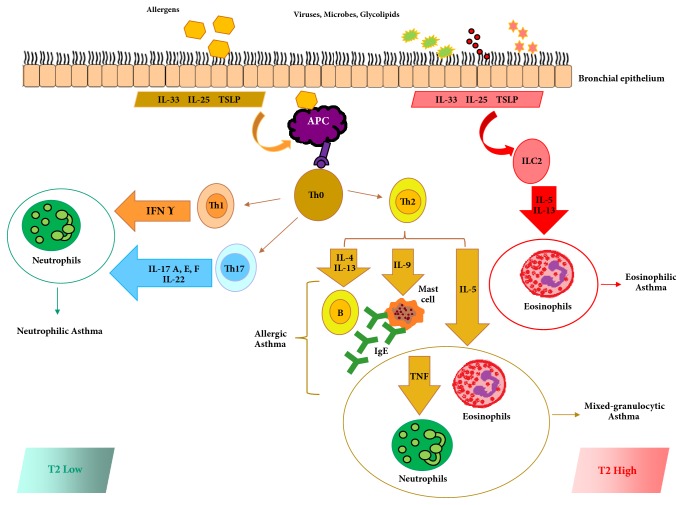
T2-high and T2-low asthma pathway. The T2-high subtype is characterized by the presences of high level of eosinophils in airways, and the T2-low subtype is characterized by Neutrophilic or Paucigranulocytic airway inflammation. APC antigen presenting cell, ILC2 type 2 innate lymphoid cells, TSLP thymic stromal lymphopoietin, and TNF (tumor necrosis factor).

The T2-low subtype is characterized by Neutrophilic or Paucigranulocytic airway inflammation and may consist of the following: 1. obesity-related asthma, late-onset; 2. asthma and chronic obstructive pulmonary disease overlap syndrome (ACO)/Neutrophilic, late-onset; 3. smoking-related asthma; 4. paucigranulocytic, associated with smooth muscle (Figure 1) [4].

## 4. T2-High

### 4.1. Pathogenesis and Potential Biomarkers

Most of the new biologic drugs target the Th2 cytokines pathway. These cytokines (IL-3, IL-4, IL-5, IL-9, and IL-13) are expressed in bronchial submucosa and could trigger the release of mediators that could support other inflammation patterns as well, such as thymic stromal lymphopoietin [6]. Type 2-high asthma involves different important inflammatory cells including type 2 innate lymphoid cells, Th2 cells, natural killer T cells, and mast cells. Cytokines contribute to the activation and recruitment of immunoglobulin (Ig) E antibody-producing B-cells, which sustain the allergic airway inflammation. Recently, McKenzie and colleagues described group 2 lymphoid cells producing these cytokines, defining another pathway which contributes to the T2 high profiling (Figure 1) [7]. At present, several biomarkers can identify inflammatory characteristics of T2-high endotypes (serum IgE, serum periostin, blood eosinophil, and exhaled nitric oxide eNO) both for adult and in children asthma (Table 1) [8]. However, the most validated method to assess airway inflammation is currently the sputum cytometry. At present, four inflammatory patterns can be defined based on the granulocytes detected in the sputum: 1. Eosinophilic, 2. Neutrophilic, 3. Mixed-granulocytic (both neutrophils and eosinophils are elevated), and 4. Paucigranulocytic (neither neutrophils nor eosinophils are elevated).

According to several studies, Eosinophilic asthma is defined by the presence of elevated sputum eosinophil count (>3% with or without degranulation) and/or blood eosinophil count (≥400 cells/*μ*L) detected at least in two consecutive controls and by symptoms and exacerbations control obtained with treatment aimed at suppressing eosinophils [9]. However, for the other three patterns, there is no indication of minimum thresholds.

### 4.2. Targeted Therapy

In recent years, the therapeutic options that target the T2-high subtype have been significantly increasing. At present, the best biomarker predicting a good response to anti-IL-5, anti-IgE, anti-IL-4/IL-13, corticosteroids, and receptor for prostaglandin D_2_ (CRTH2) is the blood eosinophils count, while periostin and dipeptidyl peptidase-4 (DDP-4) can predict response to anti-IL-13 [5].

*IgE Blockers. *Omalizumab, the first biological approved for asthma, is a humanized monoclonal antibody (mAb), approved in 2003 by US Food and Drug Administration (FDA). It depletes IgE antibodies and blocks their action on effector cells, by reducing the density of high-affinity IgE receptors [10]. Omalizumab is effective in patients aged 6–75 years with allergic asthma and sensitized to perennial allergens and present levels of IgE serum ≥30UI/mL≤1500 UI/m. Omalizumab showed efficacy and safety in randomized clinical trials (RCTs) and real-life setting, in terms of reduction of exacerbation rates and steroid-sparing effect [11]. Furthermore, it indirectly decreases airway eosinophilia and for this reason it is more effective in patients with higher levels of exhaled oxide nitric, blood eosinophils, or blood periostin [11, 12]. These combined biomarkers also showed a predictive value for clinical response, and discontinuation of anti-IgE treatment in patients with these features demonstrated a more rapid loss of asthma control [13]. Severa studies confirm that a long-term treatment with omalizumab allows an improvement of symptom control and a sustained reduction of exacerbation risk in adult patients. [13, 14]. On the opposite, there is still a lack of biomarkers that can guide the clinician in continuing or suspending treatment with patient growth in pediatric populations. However, Baena-Cagnani and coworkers showed that omalizumab may have a disease-modifying effect in children with moderate/severe uncontrolled asthma. During the first 3 years of follow-up, after the treatment with this drug, they were completely free of asthma symptoms [15].

Quilizumab, a humanized IgG1 mAb, targets the M1-prime segment of membrane-expressed IgE causing the depletion of IgE-switched and memory B-cells. Unfortunately, clinical studies did not confirm a significant clinical efficacy in patients with uncontrolled refractory allergic asthma [16]. Ligelizumab (QGE031), an IgG1k anti-IgE mAb, has a major suppressor effect on free IgE compared with the gold standard omalizumab, with a better pharmacodynamic effect in allergic subjects, even in the case of higher IgE levels. These improvements could allow a successful treatment in patients who show inadequate response or unresponsiveness to omalizumab [17].

*Anti-IL-5. *Mepolizumab and reslizumab are two mAbs that bind IL-5 with high specificity and affinity and have been recently approved for the treatment of severe uncontrolled Eosinophilic asthma. Mepolizumab is a N-glycosylated IgG1/k humanized mAb, approved as an add-on subcutaneous therapy in patients aged at least 18 years with severe Eosinophilic asthma and blood eosinophil levels of 300 cells/mcL or greater and 150 cells/mcL during the previous 12 months [18]. Mepolizumab showed efficacy in the reduction of exacerbations, it exerts an oral glucocorticoid-sparing effect, and it determines the improvement of quality of life. However, data on the increase of Forced Expiratory Volume in 1 Second (FEV1) were contradictory except in the MUSCA study [19]. Reslizumab, an IgG4/k mAb, has been approved as an add-on intravenous monthly treatment in patients aged at least 18 years with severe Eosinophilic asthma, with baseline blood eosinophilia ≥400 cells/*μ*L [20]. It improves asthma control, FEV_1_, and quality of life (QoL) [21, 22]. However, there are still concerns about the route of administration and the real positioning of this drug in the general context.

Benralizumab, an anti-eosinophil mAb approved in 2017 by FDA, is an IgG1/k antagonist of the *α* chain of human IL-5 receptor [23, 24]. This drug is the only one that can induce apoptosis by means of cellular toxicity mechanisms (antibody-dependent cell-mediated cytotoxicity or ADCC) in its target cells, reducing the level of eosinophils in tissues by 90–100. Furthermore, its clinical effect is independent of the IL-5 circulating levels, which usually tends to increase during asthma exacerbations [25]. Data from RCTs confirmed the efficacy of benralizumab in reducing annual exacerbations rates and improving FEV1. Moreover, benralizumab showed a significant systemic steroid-sparing effect [26, 27].

*Anti-IL-4, AntiIL-13. *Dupilumab, a fully humanized mAb anti-IL-4 receptor currently investigated in phase 3 studies, inhibits the biologic effect of both IL-4 and IL-13 by preventing their interaction with IL-4 receptor *α* subunit. Several studies have demonstrated its efficacy in the reduction of asthma exacerbations and improvement of symptoms, QoL, and respiratory function [28, 29] irrespective of their baseline blood Eosinophilic count. There are still doubts about its safety profile, in particular regarding the evident rise of blood eosinophil levels which happens predominantly in patients with asthma and elevated baseline serum eosinophilia [30].

Antibodies targeting free circulating IL-4 (pascolizumab, altrakincept) [31, 32] or IL-13 (anrukinzumab, IMA-026, GSK679586) have been studied and appear safe and tolerable, but they have been discontinued due to failure in reaching primary outcomes [33–35].

Lebrikizumab and tralokinumab, two IgG4 anti-IL-13 mAb binding free-IL-13, are still under development but, so far, did not show any clinical improvement in asthma exacerbation rate and only a modest clinical effect has been demonstrated [36, 37].

*Novel Therapies. *Several other drugs are currently under development, including antithymic stromal lymphopoietin (TSLP) such as AMG157-tezepelumab [38] that mitigates the early and the late-onset-phase responses to allergens. TSLP, IL-33, and IL-25 are key mediators of type-2 inflammation diseases (such as asthma, nasal polyposis, and Eosinophilic esophagitis); therefore they are deserving an increasing interest as potential target of new drugs. IL-25 and L-33 inhibitors have unfortunately not reached the clinical outcomes [39].

The stimulation of prostaglandin antagonist's receptor (CRTH2), present on lymphocytes, eosinophils, and basophils surface, induces chemotaxis of these inflammatory cells and the release of mediators. There is a growing interest in exploring drugs targeting this receptor (fevipiprant, setipiprant, and OC000459) and some results are emerging, such as the improvement in FEV_1_ demonstrated by Pettipher et al. when a specific CRTH2 antagonist was used for patients with eosinophils count > 250 cells/*μ*L [40, 41].

New interest is emerging around interferon, as it is well known that respiratory viruses, especially rhinovirus, are implicated, not only in asthma exacerbations but also in the pathogenesis of asthma and Th2 inflammation. A phase 2 RCT with IFN-*β* treatment has been shown to be effective in enhancing innate immunity both systemically and in the lung (it has been demonstrated by serum concentration of CXCL10 as well as through the improvement of morning peak-expiratory flow (PEF)) in severe asthmatic patients [42]. The nebulized IFN-*β* treatment seems to act on the viral-response pathway and, administrated at the early onset of cold symptoms, prevents worsening of asthma symptoms.

T2-high blockers are responsible for clinical benefit in many patients with T2-high asthma but this may cause recurrence of the symptoms [43], so that a true immunomodulation has not yet been demonstrated. However, there is still the need of further studies that involve larger group of patients to detect and evaluate new endotypes, particularly for patients that are unresponsive to the treatments so far available.

The modern medicine is increasingly moving towards precision therapy, because it is unlikely that one therapeutic approach will be able to offer clinical benefit to all T2-high asthmatic patients. Clinical trials will benefit from a careful assessment of the targeted pathway and this should be reached by means of molecular phenotyping approach.

## 5. T2-Low

### 5.1. Inflammatory Mechanisms

Neutrophilic asthma is characterized by elevated neutrophils (≥64%), but not eosinophils (<3%), by increased total cell count (≥9.7 million cells/g) detected at least two times, and by unresponsiveness to treatments suppressing eosinophils.

Mixed-granulocytic asthma is identified when there is evidence of both neutrophils and eosinophils on at least two detections, independently or concurrently.

Paucigranulocytic asthma has low eosinophils (<3%) and low neutrophils (<64%). In that case treatments aimed at suppressing both inflammatory patterns are ineffective in controlling symptoms [3]. On the basis of inflammatory mechanisms, two different patterns can be identified: Th1 and Th17. Th1 cells release IFN*γ*, which is involved in intracellular infections and autoimmunity. Th17 cells are CD4+ T lymphocytes expressing IL-17A, IL-17E, IL-17F, and IL-22, which can activate neutrophils through the production of IL-8 (Figure 1) [44]. Airway damage associated with Neutrophilic inflammation leads to mucus gland hyperplasia and hypersecretion, airway hyperreactivity, remodelling, and corticosteroid insensitivity [45].

Regarding airway remodelling, it can be considered as a result of an impaired mucosal repair process, characterized by increased airway smooth muscle mass, subepithelial fibrosis, and increased number of mucous glands and goblet cells caused by Th2 cytokines as well as by growth factors and cytokines produced by epithelial cells and macrophages. These structural modifications alter airway mechanism and contribute to airway hyperresponsiveness [44].

### 5.2. Pathogenesis and Treatment Options

T2-low field represents a new evolving research area, and to date there are no effective therapies. This endotype is characterized by non-Eosinophilic airway inflammation. It occurs in nearly 50% of patients with asthma [46, 47]. T2-low can be subdivided into Neutrophilic, characterized by mediators implicated in the pathogenesis of Neutrophilic inflammation, such as IL-8, IL-23, and IL-17, and Paucigranulocytic inflammation. These patients show not optimal response to corticosteroids, but they have demonstrated good responsiveness to a group of antibiotics, macrolides (azithromycin and clarithromycin). The Neutrophilic inflammation in asthma may be due to corticosteroids treatment inducing impaired apoptosis of neutrophils and Th17-mediated Neutrophilic inflammation, pulmonary infections, smoking habit or occupational exposition, and altered airway microbiome [48].

Therapies for non-Eosinophilic inflammation may include macrolides, statins, and theophylline but data are still controversial [49, 50].

Other novel small molecules targeting Neutrophilic inflammation were investigated, such as C-X-C-chemokine receptor (CXCR2) antagonists, CXCL8 (IL-8), and peroxisome proliferator-activated receptor-*γ* (PPAR*γ*) [51]. Published results are contradictory [52, 53].

As for the monoclonal antibodies brodalumab and secukinumab (both anti-IL-17A), no improving in asthma symptoms has been shown [54] and daclizumab (anti-CD-25) is effective in improving symptoms and function, but it is unclear to which patients it should be addressed [55]. Studies that investigated TNF*α* blockers (etanercept, golimumab) did not demonstrate a significant clinical effect in treated patients [56, 57]. Some other chemokines could be targeted, including IL-1*β* or IL-6 [58] FLAP (5-lipoxygenase-activating protein) inhibitors such as GSK-2190915, which prevents the formation of LTB4 [59], involved in response to allergen. No active clinical trial is currently in development.

*Phosphodiesterase (PDE) and Protein Kinase Inhibitors. *PDE4 inhibitors and dual PDE3 and PDE4 inhibitors exert immune-modulatory effect potentially effective on asthma inflammation. RPL554 is a molecule registered in a clinical trial for the treatment of asthma and COPD [60]. Proteins kinases are involved in the cellular pathway of proinflammatory cytokines. Different molecules are under development [61], including PIK3 kinase inhibitors. PIK3 inhibition partially shares the mechanisms of action of low dose of theophylline [62] and some studies have demonstrated a potential effect in restoring corticosteroid sensitivity [63].

There are no active phase 3 clinical trials for the target molecules of the T2-low type. It seems that the knowledge on the pathophysiology of this endotype is still poor and no treatments are currently available. Understanding the biology and the pathophysiology of the disease will require a closer collaboration between clinical specialists and biologists, in a multidisciplinary effort.

## 6. Bronchial Thermoplasty

Bronchial thermoplasty (BT) is a nonpharmacological endoscopic procedure based on controlled heat release. The potential effect is an appreciable change in airway wall structure, by reducing the amount of smooth muscle with a device called Alair™ Catheter (Boston Scientific, Natick, MA, USA). BT is delivered in 3 short sessions, and no incisions or full anaesthesia is necessary. Each session is routinely performed under deep sedation administered by an anaesthesiologist and typically it takes 30–40 minutes to be completed. The procedure consists in the treatment of right lower lobe, left lower lobe, and right and left upper lobe in three different sessions. Sessions are performed every three or four weeks.

BT was approved by FDA in 2010 and according to last ERS/ATS guidelines it is recommended in adults with severe refractory asthma after approval by an Institutional Review Board [64]. The mechanism of action remains unclear. The literature reports reduction of ASM as a BT target [65]. Recently our team demonstrated a reduction of nerve fibers in epithelium and ASM. This result could explain the clinical improvement of patient that underwent BT [66].

Today literature addressing BT treatment of the T2-low endotype characterized by Neutrophilic or Paucigranulocytic airway inflammation is poor [3]. In our experience, patients were included into BT pathway first in the context of a clinical trial and subsequently as a clinical practice procedure.

Patients were also enrolled for BT treatment when not responsive to mAbs. Moreover, BT may be considered as a preferential treatment for patients who could not be addressed to other therapies, or who decided to perform a once-in-a-lifetime therapy [67]. On the basis of our experiences and clinical data, BT shows long-term effectiveness [68] and, therefore, it should be considered not only an experimental procedure but rather an important treatment option for adult patients with severe asthma [69].

## 7. Biomarker Discovery: Through the Detection of Novel Endotypes

Omics are a neologism that defines a new “global” molecular biology point of view that through a single analysis can characterize large-scale members of biochemical pathways and molecular functional activities. From DNA microarray to Next Generation Sequencing (NGS), the omics sciences are increasing, and they involve new techniques and approaches in order to better understand the disease and therefore enabling more effective drugs and therapies [70].

Omics technology is providing new biomarkers that may be used as novel targets for diagnostic tests and pharmacologic treatments. This will happen through the increasing of the knowledge of biological mechanisms and the microenvironment of asthma inflammation. This path is moving forward to the development of “precision medicine” approaches [70, 71].

Omics are contributing to help precision medicine to identify the right therapy to the proper clinical phenotype. The added value of omics technologies is particularly evident in severe asthma studies aimed at identifying novel endotypes.

**Figure 2:**
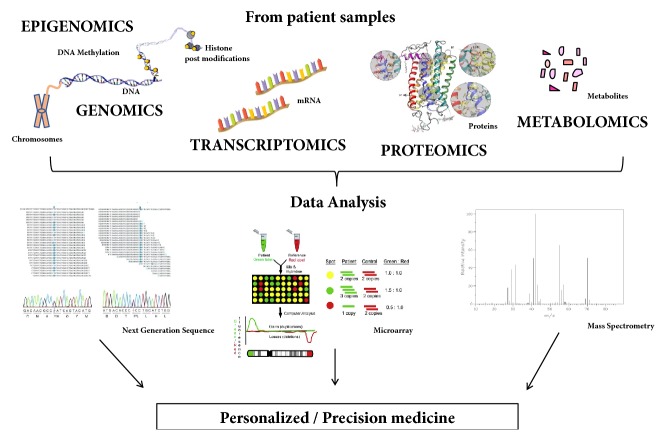
From omics technology to personalized medicine. From DNA microarray to Next Generation Sequencing (NGS), system biology provides for management and data analysis. This path is moving forward to the development of “precision medicine” approaches.

Suffix “ome” derives from “chromosome” and today includes genomics, transcriptomics, proteomics, metabolomics, and epigenomics (Figure 2).

*Genomics* is a branch of genetics that studies the sequencing and analysis of an organism's genome.

*Transcriptomics *is the study of complete set of RNA transcripts that are produced by the genome.

*Proteomics* refers to the systematic identification and quantification of the complete complement of proteins (the proteome) of a biological system (cell, tissue, organ, biological fluid, or organism).

*Metabolomics *concerns the scientific study and analysis of the metabolites produced by a cell, a tissue, or an organism.

Epigenomics is the study of all of the epigenetic changes in a cell [72].

## 8. Large-Scale Projects and the Development of Databases

To date several international projects that involve hundreds of adult and children asthmatic patients have been developing in several countries, in order to better understand severe asthma, determine differences among asthma patients, and gain new finding to create new therapeutics options.

One example of these projects is the Severe Asthma Research Program (SARP), supported by the NIH (National Heart, Lung and Blood Institute) in the United States. This program is a network enrolling over 700 patients both adults and children coming from several states [73].

Another example is the “Unbiased Biomarkers for the Prediction of Respiratory Disease Outcomes” (U-BIOPRED). This is a European project involving 16 centers in 11 European countries. The goal of this large-scale dataset is to increase the numbers and types of asthma biomarkers by integrating clinical data with inflammatory biomarkers derived from omics. U-BIOPRED provided an unbiased algorithm that trough system biology technology matches an exponential quantity of data that will enable phenotyping severe asthma and will pave the way to new tailored therapeutic approach [74, 75].

Within the European Union, national databases have been starting to develop. For example, Italian researchers have recently created the Severe Asthma Network in Italy (SANI). It is a multicenter register, which involves referral centers for the treatment of severe asthma. Up to now 549 adults and children patients with different types of asthma have been included [76, 77]. The aim of this registry is to identify and characterize patients eligible for biological treatments (biomarkers evaluation and causal-endotype identification), to evaluate cost/benefit optimization in the field of new and traditional treatments, and to investigate treatment adherence and its determinants. SANI Network provides the opportunity to create international collaborations with networks of clinical researchers, in the respiratory field, in the framework of the SHARP program (Severe Heterogenous Asthma Research Collaboration, Patient Centers) or ISAR (International Severe Asthma Registry) [78].

Many other countries have activated severe asthma registries, such as United Kingdom, Belgium, Germany, and Australia. These initiatives are providing a huge amount of information for large-scale experiments, with the hope that this data will be freely accessible [79–82].

## 9. Genomics of Asthma

Different genes have been associated with asthma severity. Genome-wide association studies (GWASs) contributed to identifying several asthma risk loci. To date, several studies have been performed trying to link the disease with portions of the genome through the use of a high number of Single Nucleotide Polymorphisms (SNPs) [83]. A major study of the GABRIEL consortium in Europe involved 10,365 patients and 16,110 healthy controls who were where genotyped of 582,892 SNPs. One of the major findings was the identification of the* ORMDL3 *(ORM1-like protein 3) and the* GSDMB *(Gasdermin-B) genes within 17q21 locus and* CDK12* (Cyclin-dependent kinase 12) as candidate genes for childhood-onset severe asthma [84]. The same 17q21 locus was also associated with asthma exacerbations, and treatment response, and* ORMDL3* was proposed as candidate gene [85].* ORMDL3 *negatively regulates the expression of IL-2. Considering the role of IL-2 in the differentiation of TH2 cell subsets, this effect on IL-2 production could represent a genetic risk for asthma and autoimmunity [86]. As a major finding of these projects, asthma onset has been associated with a number of genes coding for HLA, IL-13, IL-33, thymic stromal lymphopoietin [TSLP], IL-1 receptor-like 1 [IL-1RL1], ST2, and the receptor for IL-33. Furthermore RAR-related orphan receptor A [RORA], SMAD family member 3 [SMAD3], and GATA3 were identified [87].* CDHR3 *gene has also been described in severe asthma in addition to* GSDMB*,* IL-33,* and IL-1RL1. Furthermore,* CDHR3* gene expression was associated with exacerbation in children asthma population from 2 to 6 years of ages [88, 89].

A study conducted on severe asthma in Italian patients found a significant association between the SNP rs848 within the* IL-13* gene and severe asthma symptoms [90]. A correlation between HLA-II genes and different asthma phenotypes has also been described. In particular, HLA-DRB1 is associated with allergic asthma, HLA-DQB1 with occupational asthma, and HLA-DPB1 with aspirin-sensitive asthma [91].

## 10. Pharmacogenomics of Asthma

One of the most important applications of genomic association studies outcomes for severe asthma is pharmacogenomics. This omics science studies genetic variations influencing treatment response to the most commonly used asthma therapies. For this reason, several studies have already been successfully performed. For example, BARGE (Beta-Adrenergic Response by Genotype) trial showed that patients with an ARG16 variant of ADRB2 gene had a small decline in lung function compared to Gly16gly genotype. The response to the short-acting beta-2 agonist (SABA) therapy has been correlated with a mutation in the coding sequence of the beta-2 adrenoceptor [ADRB2] gene that causes a Glycine/Arginine substitution at position 16 of the receptor protein (Gly16Arg) [92]. In line with response to SABA, pharmacogenetic works on response to long acting beta-2 agonist (LABA) have addressed the ADRB2 gene but no effect on lung function was found [93]. However, the Arg16 variant showed an impact on LABA in pediatric population [94]. Several GWASs on inhaled corticosteroids (ICS) response in asthmatic population were performed but no clinically significant results were reported [95, 96]. Although Mosteller and coworkers [95] did not find any significant genetic markers in their study, other studies have identified a novel SNP, rs10044254, associated with both decreased expression of the F-Box and Leucine Rich Repeat Protein 7 [FBXL7] gene and improved symptomatic response to ICSs in pediatric subjects [97]. These very interesting findings suggest that there might be a specific genetic mechanism regulating symptomatic response to ICSs in children not present in adults.

On the opposite, variations of the* GLCCI1* gene, encoding for glucocorticoid-induced transcript 1 protein, have been associated with a reduction of pulmonary functions. Unfortunately, these results were not confirmed by other clinical trials. The rs2872507 SNP, which influences* ORMDL3* gene expression at locus 17q21, may be a possible marker for ICS treatment response in childhood asthma [98]. The rs2872507 T variant of this gene was found in the SHARP population and is associated with an improvement in FEV1 in asthmatic patient on ICS therapy. Many clinical trials investigated the response to leukotriene modifiers, and, despite a quantity of candidate gene, only 5-lipoxygenase (ALOX-5), transporter gene (MRP1), and ATP-binding cassette (ABCC1) transporters were found to be involved in the LTRA response [98]. Despite the efforts dedicated to the investigation of genetic variations associated with asthma, so far there is no evidence of strong association with treatment heterogeneity of response and no pharmacogenetic marker seems to be able to reach clinical relevance. Further investigations are therefore needed to identify specific genetic variation influencing treatment response and to provide new distinct asthma phenotypes.

## 11. Transcriptomics

Bigler and collaborators recently performed a whole genome expression of blood cell in asthma. In this research, 1,693 genes were differentially expressed in severe asthmatic patients [99]. Using bronchial epithelium and induced sputum samples, several case/control studies tried to define the different asthma subtypes through differential expression of messenger RNA (mRNA). Woodruff and coworkers identified the two most popular asthma subgroups, “T2-high” and “T2-low,” through the different expression of IL-5 and IL-13 transcripts in bronchial biopsies. Furthermore, epithelial expression of* POSTN *(periostin),* CLCA1 *(calcium-activated chloride channel regulator 1), and* SERP1NB2 *gene transcripts may be predictive of a Th2 driven inflammation [100]. Shikotra et al. found an upregulation of the* CEACAM6* (carcinoembryonic antigen related cell adhesion molecule 6) transcript in bronchial biopsies of asthmatic patients; it was associated with airway epithelial cells and tissue neutrophils, showing that the* CEACAM6 *expression levels could be linked to a Neutrophilic asthma phenotype [101].

In the past few years, some studies aimed at identifying a “T2 gene-based discrimination” in induced sputum samples, which is less invasive than bronchial biopsies [102]. Other studies detected seven gene transcripts [COX-2 (cyclo-oxygenase-2), ADAM-7 (disintegrin and metalloproteinase domain-containing protein 7), SLCO1A2 (solute carrier organic anion transporter family member 1A2), TMEFF2 (transmembrane protein with epidermal growth factor like and two follistatin like domains 2), TRPM-1 (transient receptor potential cation channel subfamily M member 1), and two unnamed] in bronchial brushing samples with expression levels that were moderately correlated with submucosal eosinophils [103].

Severe asthma in adults is characterized by inflammatory pathways involving mast cells, eosinophils, and group 3 innate lymphoid cells detected in induced sputum, endobronchial, and nasal brushing [104]. Baines et al. identified transcriptional inflammatory asthma phenotypes (TAPs) by studying the gene expression profile from induced sputum of adult stable asthma. Three distinct TAPs groups were identified: TAP 1 Eosinophilic, TAP2 Neutrophilic, and TAP3 Paucigranulocytic [105]. When the TAP profiles were compared with gene expression analyses of sputum, a 92% of overlap was detected.

*MicroRNAs. *MicroRNAs (miRNAs) are small noncoding single RNAs strands that regulate gene expression at the posttranscriptional level. MiRNAs are involved in all of the most important cells functions including the control of inflammatory processes; therefore numerous studies have been conducted to better understand the involvement of miRNAs in several diseases. The characterization of miRNAs and their role may represent an important tool for endotyping the complex asthma phenotype picture, when investigated in tissues whose collection is less invasive than common bronchial biopsies and induced sputum, such as peripheral blood.

MicroRNA expression in the peripheral blood has been investigated in a small study that compared seven mild asthmatics and four healthy subjects. This study detected an underexpression of microRNA 192 when study population underwent allergen inhalation challenge [106].

The interest in miRNA study as potential source of biomarkers is increasing. It has been shown in serum a differential expression of miR-1248 in asthmatic versus nonasthmatic patients and it has been demonstrated that miR-1248 is directly involved in the regulation of IL-5 transcript [107].

So far, severity of asthma had a minor impact on miRNA expression when it was evaluated on nasal biopsies of asthma patient [108]. However, a recent case-control study that investigated severe equine asthma identified 11 miRNAs differentially expressed. One of this miRNAs was the MiR-128 [109], which is part of a regulatory miRNA network and it has been already shown to be downregulated in bronchial epithelial cells of asthmatic patients. These results were confirmed by a significant increase of interleukin-6 (IL-6) and interleukin-8 (IL-8) that are associated with pathophysiology of asthma [110].

A preliminary miRNA study has also been performed for pediatric asthma (12 cases and 6 controls) that showed an upregulation of MiRNA-221 and miRNA-485-3p in asthmatic patient [111].

## 12. Epigenomics

Epigenetic mechanisms include DNA methylation, histone modifications, and noncoding RNAs, and they can control gene expression acting on DNA structure and subsequent regulation. The set of nucleic acid methylation modifications in an organism's genome is known as methylome. Several genes linked to asthma are regulated by epigenetic mechanism, such as genes involved in T-effector pathways (interferon INF-*γ*, interleukin-4 (IL-4), IL-13, and IL-17), T-regulatory pathways (forkheadbox P3 [FoxP3]), and airway inflammation (arginase [ARG]) [112].

A study on African American inner-city children identified 81 differentially methylated regions (DMRs) in peripheral blood mononuclear cells (PBMCs) related to allergic asthma. Several immune genes were hypomethylated including IL-13,* RUNX3, *and* TIGIT *[113].

Breton's group in 2011 studied the DNA methylation of specific genes and investigated biomarkers of airway inflammation. They found methylation levels of several CpG (regions with a high frequency of CpG site) loci located in promoter regions of ARG genes associated with FeNO. This finding could explain a possible role of DNA methylation in the regulation of nitric oxide production [114].

An epigenetic association between serum IgE levels and methylation at different loci using DNA from peripheral blood leukocytes demonstrated that genes annotated to these loci encode known eosinophil products. This finding suggests that methylation differed significantly in isolated eosinophils from subjects with and without asthma and high IgE levels [115].

## 13. Proteomics

The currently available literature provides several examples of detection of proteins involved in inflammatory mechanisms of asthma; they are commonly profiled using mass spectrometry. Proteome analyses research so far has been conducted in limited sample-size studies on bronchoalveolar lavage fluid (BALF) [116, 117], bronchial biopsies [118], and sputum supernatants [119, 120]. A large-scale study stratified severity of asthma relying on granulocytes inflammatory in sputum. Patients were divided into different groups: <2% or >2% eosinophils and <40% or > 40% neutrophils. Microarray data showed different inflammatory proteins between groups [118]. The SARP group was able to identify four groups of asthma from mild-moderate to severe using the protein expression level of 18 targeted cytokines [121].

Proteomics signatures have been investigated also in biopsies of omalizumab responder (OR) versus nonomalizumab responder (NOR) phenotypes after 36 months of treatment. Baseline galectin-3 expression was found in OR patients but not in NOR. Galectin-3 detection was related to an improving of respiratory function in OR and it could be considered as a potential biomarker of long-term response to omalizumab [122].

## 14. Metabolomics

In order to differentiate asthma endotypes, many studies recently suggested that a measure of metabolic profiles in different samples including exhaled breath, urine, plasma, and serum may be applied [123]. Nowadays the most attractive area of metabolomics is “breathomics” [124]. It is based on the use of an electronic nose that recognizes a profile of volatile organic compounds (VOCs) in exhaled breath and is able to discriminate asthmatics from healthy controls [125, 126].

One of the studies in the field obtained a fingerprint of VOCs for asthma atopic patients by employing mass spectrometry combined with electronic nose [127].

Another small size study (25 patients) compared the eNOSE performance to sputum eosinophils and exhaled nitric oxide (FeNO). The eNOSE was able to discriminate asthmatics from healthy subjects and to predict corticosteroid response in asthmatics [126]. Furthermore, the fingerprint identified by the eNOSE correlated with the percentage of sputum eosinophils [127, 128]. Metabolomics seems to be a promising tool in identifying asthma endotypes and biomarkers; however additional studies are needed in order to enhance the knowledge and obtain a standardized approach.

## 15. Conclusions

From the conventional definition of asthma to the inclusion of new contemporary endotypes, science has been making huge strides in the field of asthma. To date, the endotyping dichotomy between T2-high and T2-low based on inflammatory and pathophysiology pathways improved asthma management. Currently, T2-high endotype is better understood and most of new biological therapies address T2-high asthma treatment. Among biological treatments, omalizumab has a well-documented efficacy, safety, and effectiveness. However, many studies have been conducted on other drugs targeting the T2-high pathway and this includes mepolizumab (already available on the market) and reslizumab. Both target IL-5 and their long-term efficacy is now confirmed by many studies. Benralizumab and dupilumab, addressing as well Th2-high inflammation, will be available in the near future.

On the other hand, the pathogenesis and pathophysiology of T2-low endotypes remain so far unclear and treatment options specifically addressing T2-low pattern are still lacking, except for BT.

Although the knowledge concerning asthma mechanisms is increasing, biomarkers research needs to be improved, in order to identify molecules univocally selective for the appropriate therapies and predicting response to treatment. Achieving that goal is much more needed in the frame of mAbs sustainability.

A great support could come from the omics technologies. This is a new way to approach science and data analysis. Omics technologies are facilitating rapid advance in understanding the molecular details of asthma pathogenesis and pathophysiology. It implies a commercial counterpart both in pharmaceutical and in biotechnology research, aimed at offering system biology solutions to drug developers and diagnostics companies.

Although the interest in omics technologies is increasing, several limitations still restrict their wide clinical use. Indeed, none of the above-mentioned omics signatures have been translated into clinical practice. Large-scale studies and specific RCTs are necessary in order to find a real clinical utility and application of omics science as a biomarker and prognostic factor. For example, a study conducted in a cohort of 194 asthmatic patients identified 6 clinical and pathobiological clusters based on blood and induced sputum measures [129].

From the omics, circulating miRNA deserves a specific interest. Circulating miRNAs might be a noninvasive biomarker useful to diagnose and characterize asthma. Hyperlink's team studied the expression of miRNA in the blood of asthmatic patients compared with nonasthmatic patients. Their results showed a subset of circulating miRNA (miR-125b, miR-16, miR-299-5p, miR-126, miR-206, and miR-133b) expressed in patients with allergic rhinitis and asthma [130].

The power of the new miRNA's technology consists in easy sampling, exposing the patient to the lowest possible risk and the cheap and reproducible method of quantifying miRNA blood levels.

The recent Italian register, as well as the European (U-BIOPRED) and American (SARP) ones, can represent an excellent source for data and future studies. T2-low endotypes management represents the most urgent unmet need to be addressed through the use of these new technologies, which however provide a formidable support for better understanding and treating any asthma type.

We need to consider that real-life applicability of omics technologies is still far from the levels that could be expected. In the last two decades we have witnessed an exponential increase of biological therapies in oncologic fields and regarding therapies for treatment of rheumatic diseases, solid, and blood cancer. On the opposite, in asthma area from 2006 to 2017 omalizumab has been the only available mAb. Recently, both clinical and preclinical researches have literally exploded.

Indeed only in recent years have severe asthma therapeutics options expanded their potential thanks to development of new drugs.

Despite an increasing interest in omics technology, we need to take into account the fact that none of the omics signatures mentioned above has been translated into clinical practice and that it is one of the major limits. For this reason, development of large-scale studies is urgently needed. Particularly, specific Randomized Controlled Trials (RCTs) would be necessary to definitively confirm the clinical relevance of omics and reinforcing omics role in searching for new biomarkers and prognostic factors. The need for correctly selecting the right mAb for the right patient is one of the key points in severe asthma management.

The challenge in the “omics era” is to translate from bench to bedside this huge amount of data coming from the above-mentioned dataset. Translation in clinical practice through RCT is needed to emphasize omics' role in precision medicine and to predict response to treatments.

Finally, the study of the interaction between the different biomarkers will be extremely important for better understanding asthma progress and evaluating the possible negative impact of some therapies. For these purposes, the application of mathematical models that gather the interaction of the different biomarkers will provide a great help, as well as the application of machine learning approaches that will help to decide the most successful therapies. These promising fields seem to be still far from application in the asthma field, but we are confident they will be widely investigated in the upcoming years, similarly to other medical fields.

## Figures and Tables

**Table 1:** **Overview on asthma biomarkers.**

**BIOMARKER**	**ENDOTYPE**	**ACTIVATED CYTOKINES**	**ROLE IN INFLAMMATION PATHWAY**	**BIOLOGICAL AGENTS**
IgE (serum)	**T2 high**: Allergic	IL-4, IL-13 through activated Th2 cells	Binds Fc*ε*RI expressed on the surface of mast cells, eosinophils, basophils and B lymphocytes Leads to subsequent degranulation and release of mediators	Omalizumab

Eosinophils (serum and sputum)	**T2 high**: Eosinophilic	IL-5	Involved in production of reactive oxygen species, desquamation and lysis of airway epithelial cells Promote airway remodelling	Mepolizumab, Reslizumab, Benralizumab

Surrogate periostin (serum, sputum)	**T2 High: **Eosinophilic-Allergic	IL-4, IL-13	Induce an amplification and persistence of chronic inflammation of allergic diseases Involved in the process of subepithelial fibrosis in asthma patient and in airway remodelling	Lebrikizumab, Tralokizumab, Omalizumab

Exhaled nitric oxide (FeNO)	**T2 high**: Allergic	IL-4, IL-13	Useful surrogate of airways inflammation Due to increased nitric oxide production by activated bronchial epithelial cells	No biological agents, but guideline recommended therapies

Dipeptidyl peptidase 4 (DPP-4 serum)	**T2 High: **Eosinophilic	IL-13	Induces the proliferation of airway smooth muscle cells, lung fibroblasts and fibronectin production	Tralokinumab

Galectin-3 (bronchial tissue)	**T2 high**: Allergic	No target identified	Involved in eosinophil recruitment, airway remodelling and development of Th2 phenotype Early predictive biomarker of modulation of airway remodelling in severe asthma patients treated with omalizumab.	Omalizumab

Neutrophils (sputum)	**T2 low**: Neutrophilic/Paucigranulocytic	IL-8	Induce the release of O2, matrix metalloproteinase-9 (MMP-9), leukotrienes-4 (LTB-4), and platelet-activating factor (PAF)	No biological agents still available
